# Astrocytes’ Contribution to Adult Neurogenesis in Physiology and Alzheimer’s Disease

**DOI:** 10.3389/fncel.2018.00432

**Published:** 2018-11-27

**Authors:** Frédéric Cassé, Kevin Richetin, Nicolas Toni

**Affiliations:** Center for Psychiatric Neuroscience, Department of Psychiatry, Lausanne University Hospital, Lausanne, Switzerland

**Keywords:** neurogenesis, astrocytes, Alzheimer’s disease, neurogenic niche, secretion

## Abstract

Adult neurogenesis is one of the most drastic forms of brain plasticity in adulthood and there is a growing body of evidence showing that, in the hippocampus, this process contributes to mechanisms of memory as well as depression. Interestingly, adult neurogenesis is tightly regulated by the neurogenic niche, which provides a structural and molecular scaffold for stem cell proliferation and the differentiation and functional integration of new neurons. In this review, we highlight the role of astrocytes in the regulation of adult neurogenesis in the context of cognitive function. We also discuss how the changes in astrocytes function may dysregulate adult neurogenesis and contribute to cognitive impairment in the context of Alzheimer’s disease.

## Adult Neurogenesis

First serendipitously discovered by Altman and Das (1965) in the rat, the continuous generation of brain neurons throughout adulthood sparked great interest as a novel mechanism for brain plasticity. However, since adult neurogenesis was in apparent contradiction with the thought that anatomical stability was necessary for the brain’s functional stability and in particular for the long-term maintenance of memory, it was also met with great skepticism. It is not until the early 90s that the concept gained momentum, after adult neural stem cells were isolated *in vitro* from adult rat brains (Reynolds and Weiss, 1992) and the novel thymidine analog Bromodeoxyuridine (BrdU) enabled the identification of neuronal markers in newly-divided cells (Kuhn et al., 1996). It is now widely accepted that in mammals, adult neurogenesis occurs in two “canonical” areas, the subventricular zone and the subgranular zone of the dentate gyrus (Vadodaria and Gage, 2014), the latter being the focus of this review.

In humans, adult hippocampal neurogenesis was observed by BrdU (Eriksson et al., 1998), immunohistochemistry approaches [Boldrini et al., 2018, but see (Sorrells et al., 2018) for conflicting results using the same technology]. The use of C^14^ incorporation in neuronal DNA, in the context of atmospheric nuclear tests, suggests that the human dentate gyrus forms, on average, 1400 new granule neurons per day, representing a lifelong cumulated renewal of about 80% of all hippocampal granule neurons (Spalding et al., 2013).

### The Process

**FIGURE 1:**
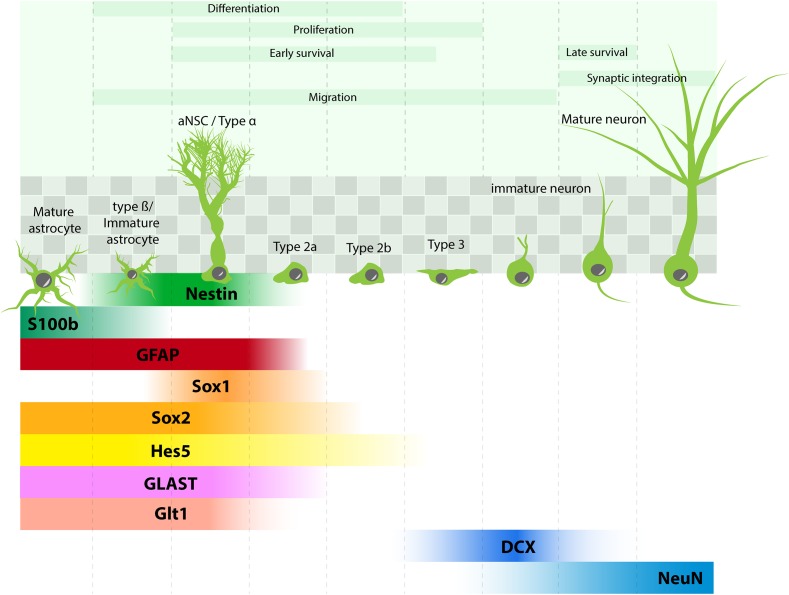
Adult hippocampal neurogenesis: process and molecular marker expression. aNSC (type α cells) are found in the subgranular zone of the dentate gyrus (shaded squares) and give rise to different progenies : type β cells (immature astrocytes), astrocytes, types 2–3 progenitors, which differentiate into neurons. Molecular markers in each cell type are depicted in comparison with markers expressed in mature astrocytes.

Recent studies using approaches borrowed from the stem cell field have uncovered the different steps of neuron generation in the adult hippocampus (Figure 1): adult neural stem cells (aNSC, also called type 1) reside in the subgranular zone of the dentate gyrus, they divide rarely and are therefore viewed as quiescent. They express stem cell markers such as Sox2, Nestin, Hes5, but also several molecular markers that are shared with astrocytes, such as the glial fibrillary acidic protein (GFAP), the glutamate aspartate transporter (GLAST), the glutamate transporter Glt1 and were even considered as astrocytes by some authors (Seri et al., 2001). In addition, their morphology is not unlike astrocytes, as they display a radial process that crosses the granule cell layer and extensively branches into the inner molecular layer of the dentate gyrus (type α cells, Gebara et al., 2016), where they establish close contacts with blood vessels and synapses (Palmer et al., 2000; Sun et al., 2015; Moss et al., 2016) (Figure 1). Clonal analysis of individual aNSC have shown that they can self-renew and give rise to progenitors, also called type 2 cells. Progenitors proliferate more actively than their mother cells and amplify several times the progenies of individual aNSC. They can be subdivided between type 2a and type 2b cells, depending on whether they still express glial or neuronal markers, respectively. Upon differentiation, neuroblast-like cells (type 3) migrate tangentially away from aNSC (Sun et al., 2015) and differentiate into granule neurons.

By virtue of the lack of proper tools to study it, the generation of astrocytes and their function in the adult brain are less well-described. Some studies find no evidence for terminal differentiation of aNSC into astrocytes (Pilz et al., 2018), while others indicate that astrocytes can be generated directly from aNSC and possibly also through type 2 progenitors (Encinas et al., 2011; Bonaguidi et al., 2012; Gebara et al., 2016).

### Role in Learning and Memory

#### New Neurons Have Increased Plastic Properties

The synaptic integration of new granule neurons into the excitatory network is a gradual process that starts at 2 weeks of cell age with the formation of dendritic spines and finishes at around 6 weeks (Laplagne et al., 2006; Toni et al., 2007, 2008). During this maturation period, new granule neurons display a lower threshold for the induction of long-term potentiation (LTP, Schmidt-Hieber et al., 2004) and an increased potentiation amplitude (Ge et al., 2007), indicative of increased plastic properties. Interestingly, the enhanced plasticity of new neurons occurs transitorily between 4 and 6 weeks, after which age the plastic properties of new neurons are similar to those of older neurons (Ge et al., 2007). Another transitory feature of new neurons is their enhanced excitation/inhibition ratio, which enables them to be highly excitable and preferentially recruited upon weak afferent activity (Marín-Burgin and Schinder, 2012). Thus, by continuously generating a population of immature neurons that are highly recruited and display enhanced plastic properties, adult neurogenesis maintains a high degree of plasticity in the hippocampal network.

#### Decreasing Adult Neurogenesis Impairs Memory Performances

Although immature neurons only represent a small fraction of all granule neurons, their strategic location in the gateway of the hippocampal formation and their preferential recruitment over more mature neurons enables them to critically influence hippocampal function. Using irradiation or cytostatic drugs, early studies found that the ablation of cell proliferation impairs LTP expression (Snyder et al., 2001), memory performances (Shors et al., 2001; Snyder et al., 2005) or pattern separation (Clelland et al., 2009). Since then, more specific approaches to inhibit adult neurogenesis have been used, which involve the controllable cell-specific expression of cytotoxic proteins (Saxe et al., 2006; Dupret et al., 2008; Imayoshi et al., 2008; Jessberger et al., 2009). However, although these studies support a role for adult neurogenesis in various memory tasks, they do not conclusively address causality. More recently, one particularly elegant approach used the optogenetic inhibition of a cohort of 4-week-old granule neurons while mice were tested for contextual fear conditioning or water maze probe trial. In both tests, memory retrieval performance was impaired exclusively upon the inhibition of the new immature neurons (Gu et al., 2012). No impairment was observed upon silencing of 2- or 8- week-old granule neurons, further supporting the age-dependency of the functional implication of new neurons. By combining the cellular specificity of the viral approach with the time precision of the optogenetic inhibition, this study clearly strengthened the role of new granule neurons in mechanisms of hippocampal-dependent memory.

#### Increasing the Formation of New Neurons Enhances LTP and Memory Performances

Of greater translational interest, several experiments aimed at enhancing memory performances by increasing adult neurogenesis. One of the environmental manipulations with the strongest effect on adult neurogenesis is voluntary exercise, which results in greater than 100% increase in newborn neuron formation, increased LTP expression in the dentate gyrus and increased memory performances (van Praag et al., 1999; Creer et al., 2010). However, given the variety of effects of exercise on general health, the role of adult neurogenesis in the running-induced memory improvements is unclear.

Recently, more specific experiments targeted newborn neurons to improve their proliferation or survival. Using the inducible cre-mediated cell-specific ablation of the pro-apoptotic gene *Bax* in nestin-expressing neural stem/progenitor cells in the adult dentate gyrus, a recent study increased the survival of new neurons by 3.6 fold in young adult mice and observed a concomitant increase in pattern separation (Sahay et al., 2011). Similarly, the inducible elimination of the endogenous Wnt antagonist Dickkopf-1 in adult neural stem/progenitor cells counteracted the age-related reduction in neurogenesis and cognitive decline (Seib et al., 2013). Finally, using the viral-mediated overexpression of the pro-neurogenic transcription factor Neurod1 in newborn granule neurons (Richetin et al., 2015) observed an increase in neurogenesis at the expense of gliogenesis, and a rescue of memory performances in a mouse model for Alzheimer’s disease (AD).

Together, these studies strongly support the view that adult neurogenesis plays a functional role in hippocampal-dependent memory. Thus, mechanisms regulating adult neurogenesis are highly relevant to cognition and especially memory.

### Role in Anxiety and Depression

Adult hippocampal neurogenesis is also very sensitive to stress, and behavioral manipulations such as unpredictable chronic mild stress, chronic social defeat, chronic immobilization and early life stress, reduce adult hippocampal neurogenesis (Anacker et al., 2018). Inversely, antidepressants increase adult neurogenesis (Surget et al., 2009). These effects are found principally in the ventral dentate gyrus, in line with a role for the ventral hippocampus in depression (Sahay et al., 2011; Kheirbek et al., 2014). Although suppressing adult neurogenesis has rarely been observed to induce depressive-like symptoms in normal mice, manipulations that reduce neurogenesis increase anxiety and depressive-like behaviors in conditions of stress, an effect that is mediated by a dysregulation of the hypothalamic-pituitary-adrenal (HPA) axis (Snyder et al., 2011). Furthermore, suppressing adult neurogenesis blocks the effect of antidepressants on several depression-like behaviors (Santarelli et al., 2003; David et al., 2010; Surget et al., 2011), suggesting that adult neurogenesis is necessary for the effect of antidepressants. Inversely, the cell-autonomous increase of adult neurogenesis mediated by the controlled knock-out of the pro-apoptosis gene Bax in adult neural stem cells reduces anxiety and depression-like behaviors in mice treated with corticosterone, a model of stress (Hill et al., 2015).

Although the mechanisms by which adult neurogenesis modulates the HPA axis, stress response and the development of depressive-like behaviors is unclear, recent results show that newborn, immature neurons in the ventral dentate gyrus inhibit the activity of stress-responsive mature granule neurons, an inhibition that is responsible for the protective effect of adult neurogenesis (Anacker et al., 2018).

Thus, it is becoming increasingly clear that adult neurogenesis plays a pivotal role in stress susceptibility and may represent a promising target for the treatment of depression.

### Regulation by the Neurogenic Niche

By definition, adult neurogenesis occurs in a mature environment and aSNC and their progenies are in contact with a variety of cells – the neurogenic niche – that can potentially influence their fate. The existence of the neurogenic niche was elegantly conceptualized by transplantation experiments from Shihabuddin et al. (2000). These authors showed that spinal cord progenitors that are restricted to glial lineage can recover neurogenic potential upon transplantation into the dentate gyrus, but not upon transplantation into the spinal cord or the non-neurogenic CA1 area of the hippocampus. These results indicate that adult progenitor cells are not lineage restricted, but can generate neurons upon exposure to appropriate environmental cues. In theory, the concept of the neurogenic niche can be extended to all biological entities that exert a regulatory role on any of the processes that occur anywhere between stem cell proliferation and new neuron functional integration. Also, the regulatory entities may in theory include all cell types of the brain as well as extracellular components, such as secreted molecules, exosomes and extracellular matrix.

Subsequently from the seminal experiment of Shihabuddin et al. (2000), numerous studies have identified extrinsic mechanisms of regulation of adult neurogenesis. However, by virtue of its embryonic-like features, adult neurogenesis is sensitive to numerous manipulations that do not necessarily regulate adult neurogenesis in a direct manner. Therefore, the identification of niche elements requires approaches that enable the unambiguous identification of cell types, their activity and their role in the regulation of adult neurogenesis. In this respect, the *in vitro* use of isolated adult hippocampal neural stem/progenitor cells enabled to test the direct effect of different cell types on adult neurogenesis. Using this approach, microglial cells were found to directly interact with stem/progenitor cells and regulate their proliferation (Sierra et al., 2010; Gebara et al., 2013). Also, the introduction of optogenetic tools enabled the identification of the mechanisms regulating of several steps of adult neurogenesis (stem cell proliferation, progenitor cell survival as well as new neurons synaptic integration (Song et al., 2012, 2013; Alvarez et al., 2016).

**FIGURE 2:**
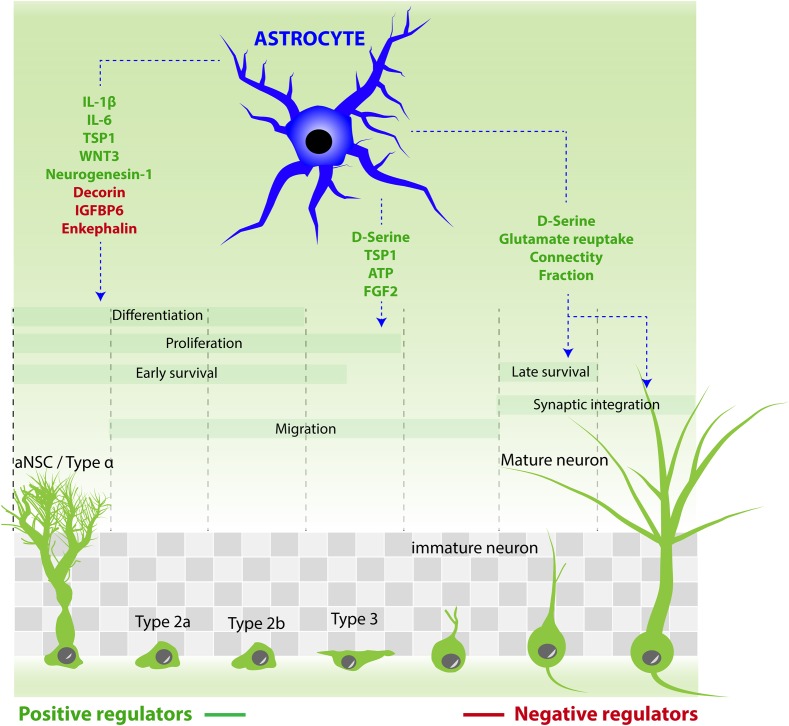
Astrocytes contribution to the different steps of adult neurogenesis by releasing active molecules. Molecules that stimulate aNSC/progenitor cell proliferation: ATP, D-serine, FGF2, TSP1. Molecules that increase neuronal differentiation: TSP1, neurogenesin-1, IL-1β, IL-6, and WNT3. Molecules that reduce neuronal differentiation: IGFBP6, enkephalin, decorin. Molecules that increase neuronal maturation and synaptic integration: D-serine. Astrocytes also regulate the synaptic integration of new neurons by reducing connectivity fraction and glutamate reuptake.

In the next paragraph, we describe the role of a major actor of the neurogenic niche, namely astrocytes (Figure 2).

## Niche Astrocytes: Structural Role

**FIGURE 3:**
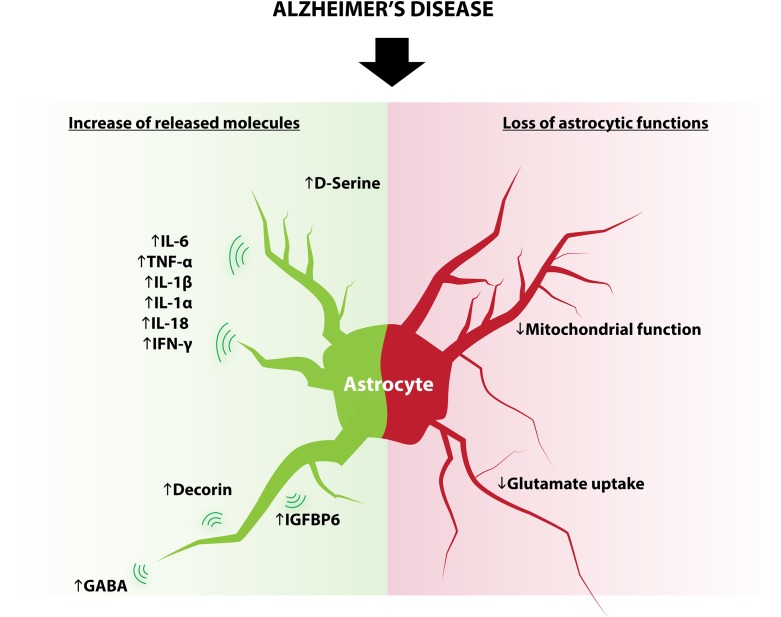
Modifications of astrocytes in AD. Some astrocytic functions known to regulate adult neurogenesis are modified in AD and include: increased expression of D-serine, IL-6, TNF-α, IL-1β, IL1-α, IL-18, IFN-γ, decorin, IGFBP6, GABA and decreased glutamate reuptake and mitochondrial function.

The principal cell type of the neurogenic niche, by number, is the astroglia. In a seminal study using dissociated and purified cells Song et al. (2002) showed that molecules produced by astrocytes can influence adult neural stem/progenitor cells, and more specifically increase their differentiation into neurons. However, although non-neuronal cells (which include astrocytes) are four times more numerous than neurons in the adult hippocampus (Fu et al., 2015), investigating their role in the regulation of adult neurogenesis has long been hampered by the lack of specific tools. In particular, the molecular similarities between astrocytes and adult neural stem cells greatly complicates genetic targeting strategies (see Box 1). Nonetheless, several studies using appropriate genetic tools and fine structural approaches have started lifting the veil on the astrocyte-stem cell communication in the physiological and in the pathological context (Figure 3).

Considerations to Study the Role of Astrocytes in Adult Neurogenesis. Since the engineering of the first transgenic mouse, the question of the cell type-specificity of transgene expression has been limited by the promoter’s strength and specificity. In studies of the role of astrocytes in the regulation of adult neurogenesis, this issue is compounded by the similarities in gene expression between astrocytes and aNSC. Indeed, aNSC express several proteins that are usually, but incorrectly, considered specific for astrocytes such as GFAP or GLAST (Figure 1) and these promoters therefore drive the expression of the protein of interest in the two cell types. Furthermore, combined to the controllable Cre-ERT2-mediated recombination system, such promoters will induce a stable recombination in astrocytes but also in aNSC and their progenies, leading to the generation of clones of neural cells (newly-formed astrocytes and neurons) that will inherit the given mutation. Therefore, a phenotype that is expected to be astrocyte-dependent may in fact reflect the transgene effect on astrocytes and on aNSC and progenies. Finally, owing to the relative fragility of aNSC and immature neurons, the main drugs used to induce transgene expression, may impair some steps of adult neurogenesis (Sultan et al., 2013; Rotheneichner et al., 2017).

Therefore, adequate strategies and adequate controls should be used to take advantage of the currently available mouse models:

*In vitro*: Using purified cells partially solves the specificity issue. Therefore, co-cultures between transgenic astrocytes and wild-type aNSC/progenitor cells enable the study of astrocyte function on adult neurogenesis.

*Timing of transgene expression*: Adult neurogenesis is a long-lasting process that, from aNSC proliferation to the full maturation of new neurons, lasts for over 6 weeks (Laplagne et al., 2006). Therefore, in order to examine the role of astrocytes on one step of this process, for example new neuron maturation, transgene expression in astrocytes can be initiated shortly after the labeling of a cohort of dividing cells. In this design, the labeled new cells are formed from wild-type aNSC, but mature in an astrocytic environment that expresses the transgene of interest.

*Promoters*: Solving the specificity issue of transgene expression will however require the discovery of novel, more specific promoters. Novel technologies such as single-cell RNA-sequencing may enable the discovery of novel promoters with increased specificity and sufficient strength to drive functionally-relevant gene expression. To this respect, the newly-generated AldH1L1-Cre-ERT2 mouse (Srinivasan et al., 2016) can be perceived as an encouraging progress, although the activity of this promoter has not been thoroughly examined in aNSC, yet.

### Astrocytes Interact With Adult Hippocampal Stem Cells

Adult neural stem cells display highly complex and specialized morphology in the dentate gyrus. With the soma nested in the subgranular zone and a fine process extending through the granule cell layer and extensively branching into the proximal part of the molecular layer, these cells have also been named radial glia-like cells. Although the function of their long and highly ramified processes is unclear, it is tempting to hypothesize that these multiple extensions enable aNSC to establish contacts with numerous cells of the dentate gyrus, which enable the fine control of their proliferation (Gebara et al., 2016).

At the ultrastructural level, nestin-GFP-expressing RGL cells observed in 3D reconstructions from serial block-face scanning electron microscopy images show multiple interactions with asymmetrical synapses, blood vessels, granule neurons and astrocytes, at the level of fine, distal processes in the molecular layer and the hilus (Moss et al., 2016). In the molecular layer of the dentate gyrus, the astrocytic processes share the blood vessel surface with processes of the stem cells, their membranes are of complementary thicknesses, and adhesion points exist between them. The direct cellular interactions between astrocyte and stem cells have been shown to be important to promote neuronal differentiation of adult aNSCs in the SVZ (Lim and Alvarez-Buylla, 1999) or *in vitro* through a juxtacrine signaling by astrocytic secretion of ephrin-B2, and activation EphB4 receptors on the stem cell (Ashton et al., 2012). On the other hand, astrocytes negatively regulate neurogenesis by astrocyte–stem cell contact mediated by Notch signaling that depends on the intermediate filament proteins GFAP and vimentin (Kinouchi et al., 2003; Wilhelmsson et al., 2012).

Thus, astrocytes are in close contact with aNSC and their effect on proliferation or differentiation depends on astrocytes’ state and protein expression profile.

### Astrocyte-Synapse Interactions on Newborn Neurons

A given astrocyte has many processes emanating from its cell body that can contact other cells, 1000s of synapses, as well as blood capillaries (Haydon and Carmignoto, 2006). During embryogenesis, nascent synapses are ensheathed by astrocytic perisynaptic processes, which are membrane specializations that express glutamate transporters and the machinery for releasing molecules (Araque et al., 2014). The shape of perisynaptic processes can be modified, depending on the structural changes of these synapses, such as induced by neuronal activity (Genoud et al., 2006). In the adult brain, these structural modifications are limited in their amplitude and seem to affect primarily microdomains surrounding synapses.

In the context of adult neurogenesis however, astrocytes of the dentate gyrus would be expected to express greater structural plasticity to accommodate the continuous incorporation of new neurons and their 1000s of dendritic spines. Indeed, the analysis of the formation of perisynaptic processes on newborn neurons showed that pre-exiting astrocytes reshape their processes to ensheathe afferent and efferent synapses of adult-born neurons (Krzisch et al., 2015). Furthermore, the blockade of astrocytic glutamate transporters induces a reduction of excitatory post-synaptic potentials and increased paired-pulse ratio on newborn neurons, indicating that functional perisynaptic processes strengthen excitatory synaptic transmission on these cells. Thus, the formation of new neurons and the establishment of their synapses induce drastic remodeling of astrocytes which, in turn, enhance their synaptic input.

Furthermore, pre-existing astrocytes seem to be involved in the choice of synaptic partners of the maturing neurons. Indeed, the dendritic spines of newly-formed granule neurons synapse with pre-existing axon terminals (Toni et al., 2007). Using serial section electron microscopy and the examination of the 3D space around nascent spines (Krzisch et al., 2015) found that new dendritic spines are surrounded by five axon terminals on average, all of which are potential presynaptic partners. Thus, the connectivity fraction, which is the number of synapses divided by the number of potential synapses is 1/5, similarly to other brain areas (Escobar et al., 2008). However, since pre-existing axon terminals are partially ensheathed by astrocytic perisynaptic processes, the intercalation of glia between nascent spines and pre-existing axon terminals potentially reduces the connectivity fraction. In the dentate gyrus, factoring the glia reduced the connectivity fraction to 1/2.9 (Krzisch et al., 2015). Finally, the expression of glutamate transporters on perisynaptic processes increase the amplitude of post-synaptic currents in newborn neurons, indicating that astrocytes regulate synaptic transmission on new neurons, once synapses are formed (Krzisch et al., 2015).

Thus, pre-existing astrocytes, by ensheathing axon terminals that may be contacted by new granule neurons, possibly interfere with the connectivity of nascent spines. Once synapses are formed, glutamate transporters on the perisynaptic processes enhance synaptic transmission of the newly-formed synapses.

## Niche Astrocytes: Secreted Molecules

The idea that astrocytes are secretory cells is almost as old as their discovery (Held, 1909) and astrocytes are now known to communicate with neurons and other glial cells by secreting a myriad of molecules such as gliotransmitters, neuromodulators, trophic factors, and hormones (Araque et al., 2014). Some neuroactive molecules produced by astrocytes may also exert either a positive or a negative action on different steps of adult neurogenesis in physiological conditions. Here, we highlight molecules produced by astrocytes that may affect (positively or negatively) the different steps of adult neurogenesis and in paragraph 4, we discuss how their gain or loss of function could contribute to the decrease of adult neurogenesis in the context of AD.

### Regulation of Proliferation

The release of neuroactive molecules from astrocytes is mediated by several distinct pathways such as channels, transporters and regulated exocytosis. The latter can be blocked using the controlled expression of either a dominant-negative form of synaptobrevin-2 or a tetanus toxin selectively in astrocytes. With this approach, the group of Cao et al. (2013a), showed that adenosine 5′-triphosphate (ATP) released from astrocytes increases the proliferation of aNSC *in vitro* via P2Y1-PLC-phosphatidylinositol 3-kinase (PI3K) signaling. Interestingly, in these transgenic mice, a lack of inositol 1,4,5-trisphosphate receptor type 2 and the block of vesicular release induce a failure in astrocytic ATP secretion, causing depressive-like behaviors that can be rescued with ATP administration (Cao et al., 2013b). Similarly, the *N*-methyl-D-aspartate receptor (NMDAR) co-agonist D-serine, a gliotransmitter known to be released by astrocytes (Mothet et al., 2005; Papouin et al., 2017), enables the proliferation of stem/progenitor cells *in vitro* (Sultan et al., 2013).

The fibroblast growth factor-2 (FGF2) is mainly produced by astrocytes and acts as a proliferative-inducing factor of aNSC (Palmer et al., 1995; Kirby et al., 2013). The idea that astrocyte-derived FGF2 regulates aNSC proliferation in physiological conditions is further supported by the age-related decline of FGF2 expressed by astrocytes observed in old mice, which is correlated with decreasing neurogenesis in the aged hippocampus (Shetty et al., 2005).

In addition to soluble molecules, astrocytes also secrete extracellular vesicles such as exosomes (Verkhratsky et al., 2016). Exosomes are generated by invagination and fission of an endosomal membrane that give rise to the intraluminal vesicle (ILV) of a multivesicular body. After fusion with the plasma membrane, ILVs release 40–100 nm diameter extracellular spherical vesicles, the exosomes (Théry, 2011). Exosomes contain a complex molecular mixture that include, cytokines, proteins, lipids, mRNA, and microRNA (miRNA) that may be biologically active in the target cell. Although it is currently unclear whether astrocyte-derived exosomes regulate adult neurogenesis, several miRNAs that are expressed in astrocytes’ exosomes are known to regulate adult neurogenesis (Jovičić and Gitler, 2017). For example, the overexpression miR-25, miR-184, miR-34a, and miR-543 in aNSC increases their proliferation (Liu et al., 2010; Agostini et al., 2011; Brett et al., 2011; Rago et al., 2014). Moreover, miR-92a inhibits the transition from aNSC cells to intermediate progenitors, suggesting a role of miR-92a in maintaining aNSC in a proliferative state (Nowakowski et al., 2013). Interestingly, the miRNAs contained in astrocytes’ exosomes are considerably different from miRNAs detectable in astrocytes, suggesting a selection of specific miRNAs for targeting exosome (Jovičić and Gitler, 2017).

### Regulation of Differentiation and Maturation

In addition to an effect on proliferation, astrocyte-secreted molecules may also modulate other steps of adult neurogenesis, such as migration and differentiation of progenitors into neurons, or the maturation, synaptic integration, and survival of newborn neurons. The first study aimed at examining the role of astrocytes in hippocampal adult neurogenesis (Song et al., 2002) showed that *in vitro*, cell culture medium conditioned by astrocytes increase aNSC differentiation into neurons. Since then, several molecules have been discovered that can participate to this effect. For example, the astrocyte-derived soluble factor thrombospondin-1 (TSP1), known for its antiangiogenic activity and for promoting synaptogenesis during brain development (Christopherson et al., 2005), also increases aNSC proliferation and neuronal differentiation *in vitro* whereas adult mice deficient in TSP1 exhibit reduced proliferation of aNSC (Lu and Kipnis, 2010). Another secretory factor, neurogenesin-1, which is produced by both astrocytes and granule neurons, increases neuronal fate of newly-formed hippocampal cells (Ueki et al., 2003). Also, IL-1β and IL-6 promote neuronal differentiation of aNSC/progenitor cells *in vitro* (Barkho et al., 2006), whereas insulin-like growth factor binding protein 6 (IGFBP6), enkephalin and decorin, also produced by astrocytes, reduce neuronal differentiation of aNSC/progenitor cells *in vitro* (Barkho et al., 2006).

Similarly to the observations of the perinatal development, astrocyte-secreted molecules also affect synapse formation on new neurons generated in the adult hippocampus: by using two independent transgenic approaches to block vesicular release from astrocytes, D-serine released by astrocytes was shown to control the dendritic maturation and the functional integration of newborn hippocampal neurons (Sultan et al., 2015). Interestingly, since both approaches only affected 50% of astrocytes (i.e., only 50% of astrocytes expressed the transgene, due to the activity of the GFAP or GLAST promoters), it was possible to observe that dendritic spine density was reduced exclusively in dendritic segments that crossed the territory of blocked astrocytes, but developed normally in the segments of the same dendrites that crossed the territories of astrocytes that did not express the transgene. This shows that astrocytes provide a local control of dendritic spines formation and suggests that astrocytes enable the integration of new neurons in local networks of increased demand for computational power.

Further substantiating the functional role of the regulation mechanisms of adult neurogenesis by astrocytes, some experiments showed that the molecular pathways involved are relevant for learning and memory: the signaling protein Wnt3 promotes neuronal differentiation of hippocampal aNSC *in vivo* (Lie et al., 2005). Inversely, the lentiviral-mediated expression of a dominant-negative form of Wnt in the adult hippocampus reduces neurogenesis and impairs spatial memory (Jessberger et al., 2009), suggesting that the astrocytic control of Wnt-induced neurogenesis is involved in the functional role of adult neurogenesis.

Thus, astrocytes control adult neurogenesis at multiple levels, from proliferation to functional integration, and provide a regulation mechanism that may have functional relevance for learning and memory, as well as for depression.

## Niche Astrocytes in Alzheimer’s Disease

Alzheimer’s disease is a chronic, progressive, multifarious, neurodegenerative disorder of aging characterized by cognitive dysfunction, including memory loss, language difficulties, psychiatric symptoms and behavioral disturbances. While AD is the most common neuropathological substrate of dementia, only few studies have examined the status of adult neurogenesis in the human AD brain. Although some reports provide conflicting data, a low endogenous proliferative activity persists in the dentate gyrus of AD patients albeit without producing functional newborn neurons (Jin et al., 2003; Boekhoorn et al., 2006; Li et al., 2008). Similarly, hippocampal cell proliferation decreases (but nonetheless persists) in several AD transgenic mice models and is followed by impaired differentiation or maturation of new neurons, resulting in altered production of functional newborn neurons (Krezymon et al., 2013; Richetin et al., 2015, 2017a,b). Indeed, retroviral targeting and identification of dividing cells in the dentate gyrus shows that 84% of dividing cells produce glial cells in 9 month old in APPxPS1 mouse (Richetin et al., 2015, 2017b).

Although microglia, the major immune cell type in the brain parenchyma, are known to play an important role in neuroinflammation, including AD, and to reduce adult neurogenesis (Sierra et al., 2010), relatively little is known on the role of astrocytes in the etiology of AD and virtually nothing on their potential participation to the impairment of adult neurogenesis in this disease. Originally, disease or lesion was described as inducing a reactive astrocyte phenotype, defined by increased GFAP expression. More recently, transcriptomic analyses enabled the finer distinction between astrocytic subtypes and their reactivity in different pathological conditions. Notably, neuroinflammation has been found to alter the expression of genes involved in synaptic transmission or in the effects of neurotrophic factors (Liddelow and Barres, 2017). Since astrocytes are of great importance for several steps of adult neurogenesis and undergo several alterations in the course of AD, we can hypothesize that both structural and molecular changes might interfere with adult neurogenesis.

### Astrocytes Structural Changes in Alzheimer’s Disease

In both patients and mouse models, astrocytes rapidly become reactive and undergo molecular, cellular, and morphological modifications in response to injury (Verkhratsky et al., 2010; Liddelow and Barres, 2017). For example, in the human hippocampus, GFAP (the major intermediate filament of astrocytes) is differentially regulated around neurofibrillary tangles and amyloid plaques (Porchet et al., 2003). In addition, astrocytes of the dentate gyrus display longer and more ramified GFAP processes and various GFAP isoforms are upregulated near amyloid plaques (Kamphuis et al., 2012, 2014). Interestingly, in the hippocampus of AD mice models, the hypertrophy of reactive astrocytes encircling neuritic plaques contrasts with the atrophy of astrocytes located at a distance from them (Kamphuis et al., 2012, 2014). This atrophy (also known as clasmatodendrosis) and astroglial degeneration is also a common phenomenon observed in several aging-related tau astrogliopathies associated with dementia (Kovacs et al., 2017).

Although it has not been demonstrated yet, it is tempting to hypothesize that the astrocytes’ loss of structure (whether hypertrophy or hypotrophy) in the dentate gyrus may impair the support they provide to neural stem cells or newborn neurons. For example, the vascular niche regulates adult neurogenesis by providing trophic factors (Licht and Keshet, 2015), a structural scaffold for neuroblasts migration (Sun et al., 2015) and by maintaining the blood–brain barrier (Otsuki and Brand, 2017). Together with pericytes, endothelial cells and their basal lamina, astrocytic endfeet participate to the integrity of the blood–brain barrier by forming a sheathing network around the brain vasculature known as the glia limitans (Ransom et al., 2003). In AD, the hypertrophy or atrophy of astrocytes impair the gliovascular unit (Kimbrough et al., 2015), which may participate to the degradation of the blood–brain barrier (Zenaro et al., 2017) and in turn disrupt several steps of adult neurogenesis such as stem cell proliferation and progenitor migration.

Another example is the integrity of gap junctions: modifications of intercellular communication between astrocytes include changes in connexin expression (Yi et al., 2016, 2018; Ariazi et al., 2017). In the hippocampus, astrocytes display an enrichment in CX43 in the vicinity of Aβ plaques in mouse models as well as in post-mortem human brain tissue (Nagy et al., 1997; Koulakoff et al., 2012). Moreover, elevated connexin immunoreactivity is also associated with plaques exhibiting bulb-like structures identified by their content in phosphorylated tau (Koulakoff et al., 2012). In normal mice, the double knockout for *Cx30* and *Cx43* in GFAP-expressing cells display an almost complete inhibition of proliferation and a significant decline in numbers of radial glia-like cells and granule neurons (Kunze et al., 2009). Furthermore, the virus-mediated ablation of connexins in proliferative aNSC was shown to reduce neurogenesis, suggesting that connexins expression by aNSC is required for proper neurogenesis (Kunze et al., 2009). Thus, AD-associated impairments of CX43 in astrocytes located in the dentate gyrus likely affects adult neurogenesis.

Since astrocytes finely regulate adult neurogenesis, their modifications associated with AD, or potentially several other brain diseases, may contribute to the impairment of adult neurogenesis that is associated with these diseases. In turn, the imbalance between neurodegeneration and neuroregeneration may aggravate disease symptoms.

### Secretome and Metabolism of Astrocytes in AD

Although in physiological conditions astrocytes produce several molecules that positively regulate adult neurogenesis, the changes in the astrocyte secretome and metabolism that occur in disease generate opposite effects. These changes include (but are not limited to) cytokines, neurotransmitters, and metabolism, all of which play an important role in the regulation of adult neurogenesis (Figure 3).

#### Cytokines

Astrocytes react to neuronal stress or injury by producing a number of molecules, including metabolites, cytokines and neurotrophins (Eddleston and Mucke, 1993; Wyss-Coray and Mucke, 2002; Nakagawa and Schwartz, 2004). Cytokines produced by astrocytes in the context of inflammation include IL-6, TNF-α, IL-1β, IL-1α, IL-18, and IFN-γ (Iosif et al., 2006; Wu et al., 2012; Choi et al., 2014) several of which are known to reduce aNSC proliferation, neuronal differentiation and survival of adult newborn neurons. For example, transgenic mice that overexpress the proinflammatory cytokine IL-6 from astrocytes show reduced progenitor cell proliferation (Vallières et al., 2002). Similarly, astrocyte-produced IGFBP6, which inhibits neuronal differentiation *in vitro* (Barkho et al., 2006), is elevated in the serum of AD patients (Tham et al., 1993); and decorin, which also decreases neuronal differentiation of aNSC/progenitor cells *in vitro* (Barkho et al., 2006), is increased in AD mice model (Lam et al., 2011).

#### Neurotransmitters

Astrocytes are also capable of releasing neurotransmitters, a process called “gliotransmission.” Similarly to neurons, astrocytes display the necessary machinery for transmitter packaging and release (Liu et al., 2011). Astrocytes release a variety of signaling molecules including glutamate, D-serine, GABA, glycine, and ATP in a regulated manner (Araque et al., 2014). Astrocytes also express transporters that enable the regulation and reuptake of extracellular neurotransmitters such as glutamate (Danbolt, 2001), GABA (Boddum et al., 2016), or dopamine (Takeda et al., 2002). The release of neurotransmitters by astrocytes undergoes significant changes in AD: reactive astrocytes surrounding Aβ deposits show an increased synthesis and release of GABA (Assefa et al., 2018), which has been shown to impair neuronal plasticity in AD mouse models (Jo et al., 2014). Furthermore, observations of AD human post-mortem brain tissue indicate a reduction of the principal astrocytic glutamate transporter EAAT2, which is consistent with reduced glutamate reuptake (Scott et al., 2011). Also, mice models for AD show a decrease of glutamate synthase expression in astrocytes, which may compromise glutamate homoeostasis (Kulijewicz-Nawrot et al., 2012). *In vitro*, application of amyloid beta to hippocampal astrocytes elevates intracellular calcium concentration through alpha seven nicotinic receptor and increases the frequency of NMDAR-mediated slow inward currents (SICs), indicating an increase in glutamate release (Pirttimaki et al., 2013). Finally, D-serine level is elevated in the hippocampus and parietal cortex of AD patients as compared to control subjects (Madeira et al., 2015). Together, these studies show that impaired astrocytic function in the course of AD contributes to the excitatory/inhibitory imbalance observed in this disease. In turn, neurotransmitter imbalance is very likely to dysregulate adult neurogenesis.

#### Metabolism

Astrocytic mitochondria buffer intracellular calcium and regulate oxidative stress and ATP production, which play a crucial role in synapse homeostasis (Liesa et al., 2009; Albrecht et al., 2011; Song et al., 2014; Kubik and Philbert, 2015). In addition, astrocytes protect neurons by buffering excessive reactive oxygen species (ROS) (Cassina et al., 2008). Although not investigated yet, these homeostatic properties likely regulate adult neurogenesis. Alterations of neuronal mitochondria have long been described in the early stages of AD, but several evidence indicate that astrocytic mitochondria are also targeted by molecular hallmarks of AD, such as amyloid beta and amyloid precursor protein (Schmidt et al., 2008; Sarkar et al., 2014). Furthermore, proinflammatory stimuli induce transient alteration of mitochondrial system in astrocytes (Motori et al., 2013). The contribution of these astrocytic modifications in AD progression is still unclear, but recent findings indicate that intrahippocampal astrocytic mitochondrial heterogeneity may contribute to progression of mild cognitive impairment through the upregulation of heme oxygenase-1 and association of astrocytic mitochondria with age-related protein inclusions (Song et al., 2014). Although it is unknown whether aNSC or newborn neurons also undergo mitochondria alterations, it is likely that the mitochondria alterations in astrocytes that occur in AD, also impair adult neurogenesis, thereby further worsening disease symptoms.

## Conclusion

Although it is still unclear whether astrocytes of the dentate gyrus represent a special class of astrocytes that enable adult neurogenesis to occur, these cells greatly contribute to the regulation of virtually all steps of adult neurogenesis, from aNSC proliferation, to the functional integration of new neurons. Interestingly, at least some aspects of this regulation seem to occur at a cellular level and be restricted to the territory of individual astrocytes. Since individual astrocytes integrate the function of the 1000s of synapses they encompassed, they are strategically positioned to control the formation and integration of new neurons in specific places of the network with increased computational demand. Thus, indirectly, these cells contribute to the required plasticity for normal hippocampal function, which is crucial for cognition. On the other hand, since astrocytes are affected by neurological diseases, such as AD, their dysfunction may impair adult neurogenesis, which possibly worsens the disease phenotype, or inhibits coping mechanisms.

In the near future, novel tools with increased specificity to target astrocytes without affecting aNSC will enable the discovery of new regulation mechanisms of adult neurogenesis and may enable the stimulation of this process in the view of brain repair.

## Author Contributions

All authors listed have made a substantial, direct and intellectual contribution to the work, and approved it for publication.

## Conflict of Interest Statement

The authors declare that the research was conducted in the absence of any commercial or financial relationships that could be construed as a potential conflict of interest.
